# Supplementary figures and images for: Identification of Unique Genetic Biomarkers of Various Subtypes of Glomerulonephritis Using Machine Learning and Deep Learning
Source: Biomolecules. 2022 Sep 10;12(9):1276. doi: 10.3390/biom12091276 (PMC9496457; doi:10.3390/biom12091276)

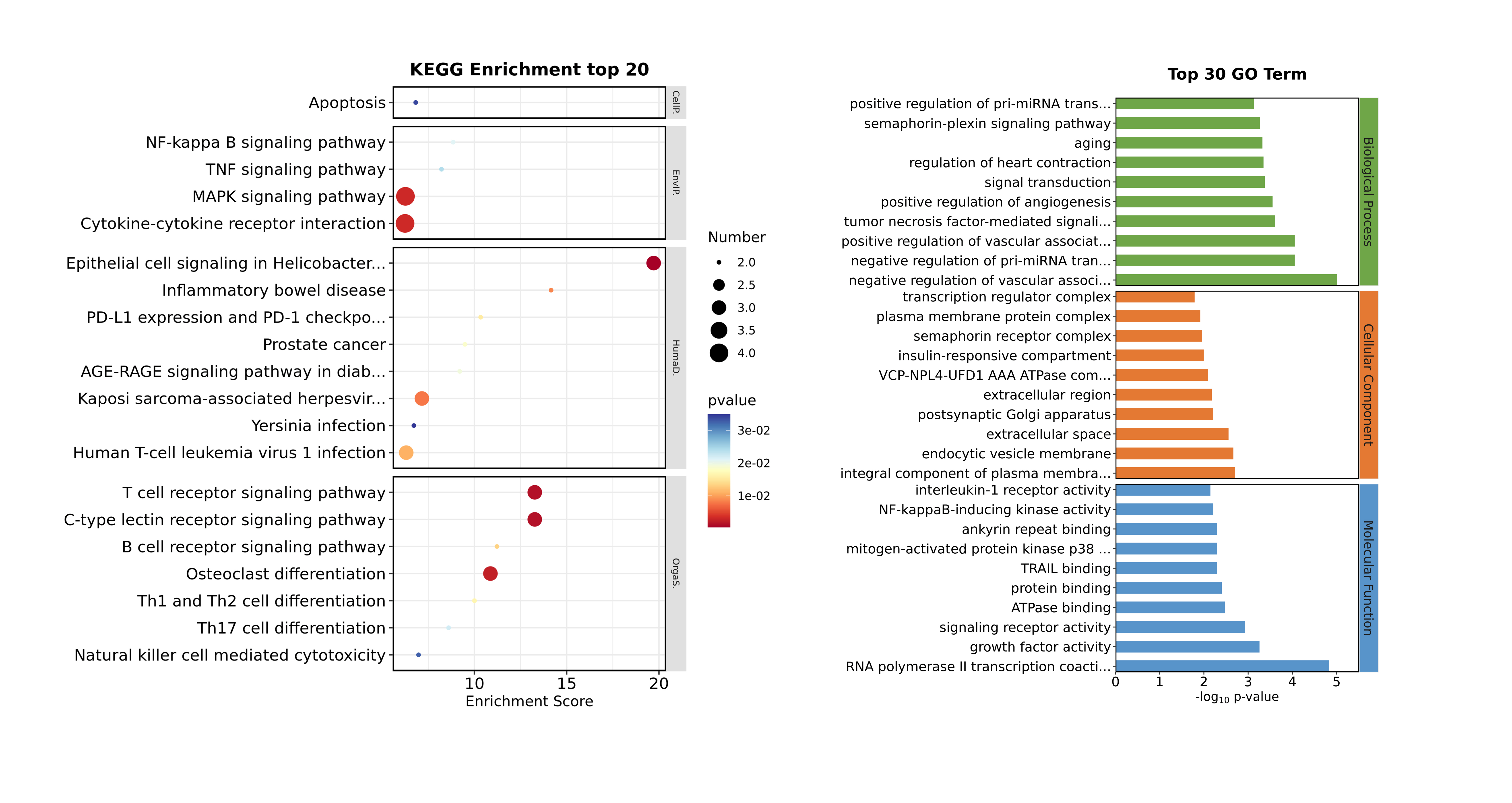

Supplement: Supplementary file 1 [file biomolecules-12-01276-s001.zip › biomolecules-1894402-supplementary final/Supplementary Figure 2.jpg]

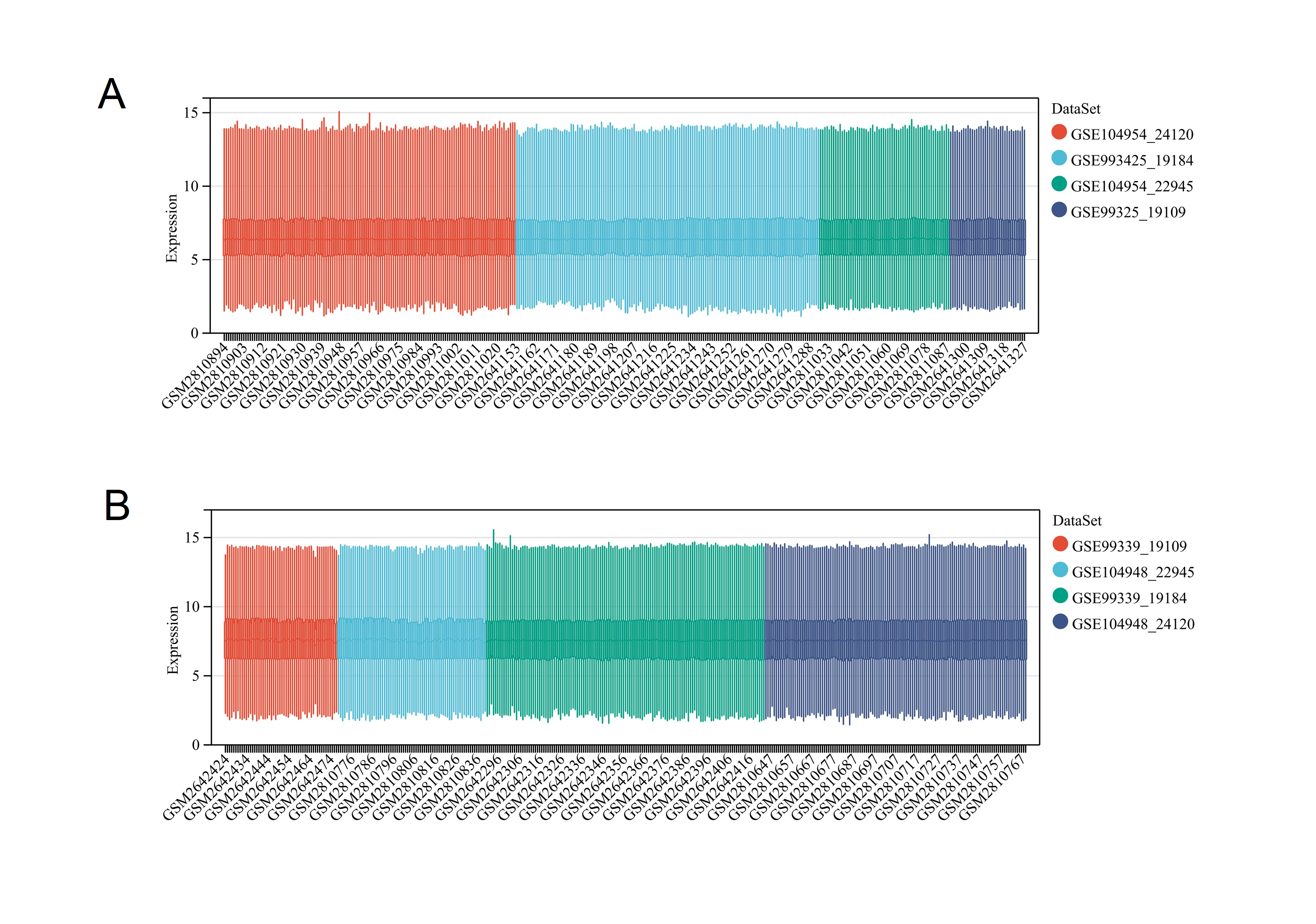

Supplement: Supplementary file 1 [file biomolecules-12-01276-s001.zip › biomolecules-1894402-supplementary final/Supplementary figure1.jpg]
